# Genomic sequencing for newborn screening: current perspectives and challenges

**DOI:** 10.3325/cmj.2024.65.261

**Published:** 2024-06

**Authors:** Nidhi Shah, Petar Brlek, Luka Bulić, Eva Brenner, Vedrana Škaro, Andrea Skelin, Petar Projić, Parth Shah, Dragan Primorac

**Affiliations:** 1 Dartmouth Health, Lebanon, NH, USA; 2 St. Catherine Specialty Hospital, Zagreb, Croatia; 3 Greyledge Europe Ltd, Zagreb, Croatia; 4 International Center for Applied Biological Sciences Ltd (ICABS), Zagreb, Croatia; 5 Eberly College of Science, The Pennsylvania State University, State College, PA, USA; 6 Medical School, University of Split, Split, Croatia; 7 Medical School, Josip Juraj Strossmayer University of Osijek, Osijek, Croatia; 8 The Henry C. Lee College of Criminal Justice and Forensic Sciences, University of New Haven, New Haven, CT; 9 Regiomed Kliniken, Coburg, Germany; 10 Medical School, University of Rijeka, Rijeka, Croatia; 11 Faculty of Dental Medicine and Health, Josip Juraj Strossmayer University of Osijek, Osijek, Croatia; 12 Medical School, University of Mostar, Mostar, Bosnia and Herzegovina; 13 National Forensic Sciences University, Gandhinagar, India

## Abstract

Traditional newborn screening (NBS) serves as a critical tool in identifying conditions that may impact a child's health from an early stage. Newborn sequencing (NBSeq), the comprehensive analysis of an infant's genome, holds immense promise for revolutionizing health care throughout the lifespan. NBSeq allows for early detection of genetic disease risk and precision personalized medicine. The rapid evolution of DNA sequencing technologies and increasing affordability have spurred numerous endeavors to explore the potential of whole-genome sequencing in newborn screening. However, this transformative potential cannot be realized without challenges. Ethical aspects must be carefully navigated to safeguard individual rights and maintain public trust. Moreover, genomic data interpretation poses complex challenges due to its amount, the presence of variants of uncertain significance, and the dynamic nature of our understanding of genetics. Implementation hurdles, including cost, infrastructure, and specialized expertise, also present barriers to the widespread adoption of NBSeq. Addressing these challenges requires collaboration among clinicians, researchers, policymakers, ethicists, and stakeholders across various sectors. Robust frameworks for informed consent, data protection, and governance are essential. Advances in bioinformatics, machine learning, and genomic interpretation are crucial for translation into actionable clinical insights. Scalability and improving downstream health care access are vital for equitability, particularly in underserved communities. By fostering interdisciplinary collaboration, advancing technology and infrastructure, and upholding ethical principles, we can unlock the full potential of NBSeq as a tool for precision medicine and pave the way toward a future where every child has the opportunity for a healthier, genomics-informed start to life.

Traditional newborn screening (NBS) has long been a cornerstone of pediatric health care, facilitating the early detection of early-onset conditions and enabling timely interventions to improve outcomes (1,2). Several disorders that would benefit from presymptomatic identification are not amenable to traditional NBS (3). However, the advent of newborn sequencing (NBSeq) heralds a paradigm shift in newborn screening, offering comprehensive genomic analysis with the potential for precision medicine tailored to individual genetic profiles (4,5). Many recent studies have aimed to explore the various aspects of integrating genomic sequencing into newborn screening. Careful considerations must be made regarding its effectiveness, utility, cost-benefit, and ethical and social aspects. This manuscript critically examines the transformative potential of NBSeq, delineating its benefits and addressing the multifaceted challenges it faces. By elucidating these complexities, we aim to pave the way toward the widespread adoption of NBSeq that would ensure equitable access to precision pediatric health care.

## The current state of newborn screening and newborn sequencing

Population-based NBS has been widely recognized as a pivotal public health program. However, the implementation of NBS in terms of the number of disorders varies from country to country and indeed even from state to state. The United States Secretary of Health and Human Services’ Advisory Committee on Heritable Disorders in Newborns and Children has recommended the inclusion of 32 conditions on its Recommended Uniform Screening Panel, which have been adopted by all states, with some state-to-state variability. One of the keys to the success of NBS programs has been the timing at which NBS is deployed, ie, the first few hours to days of life. Pre-symptomatic treatment of screen-positive newborns has been much more cost-effective and less burdensome on health care systems than treating after symptoms appear (6). Parents/guardians of children screened with a positive NBS reported less difficulty in receiving needed specialist care as compared with parents/guardians of children with genetic conditions who were diagnosed later (7).

With the rapid development of genomic sequencing technologies and the continuous improvement of their diagnostic capabilities, researchers worldwide are studying the feasibility of expansion of NBS through NBSeq (8-13).

When evaluating the utility of genomic newborn screening results, certain metrics are useful as they demonstrate the impact of timely screening and subsequent diagnostics. Rare genetic diseases are a significant cause of infant mortality. From the cohort that experienced infant mortality due to genetic disorders, 62% of infants had no previous concern for a genetic cause and 30% of diseases had outcome-improving treatments, which indicates that genomic screening early in life could significantly affect infant mortality (14). NBSeq has the potential to reduce infant mortality by identifying infants with treatable genetic disorders at the earliest stage prior to the onset of irreversible symptom progression. This is an especially important consideration for many genetic disorders that do not have biochemical alterations detectable by traditional NBS since they are most manageable in the pre-symptomatic stage (3,15). Furthermore, Green et al assessed the actionability of monogenic disorders detected in their cohort of newborns randomized to receive NBSeq with analysis for childhood-onset, childhood-actionable, and adulthood-actionable conditions. By using a modified ClinGen actionability metric, all detected disorders were demonstrated to have moderate to high actionability (8).

Most disease experts also recognize the benefit of NBSeq. Gold et al surveyed rare-disease experts on their attitudes and beliefs regarding NBSeq, as well as which gene-disease pairs should be considered for screening. A majority of experts agreed that treatable monogenic disorders should be a part of the screening program, even if the variants have low penetrance, while a minority agreed that actionable adult-onset conditions and conditions without therapeutic or management guidelines should be included. Lists of 25, 42, and 432 genes (with each list including the previous) were endorsed by 85%, 80%, and 50% of experts, respectively (16).

While the clinical perspectives on newborn screening provided by expert clinicians are of great value, the viewpoints of the general public and regulatory entities must also be taken into account. A key aspect of implementing new practices into medicine is ensuring the public is well-informed about the procedure. This, in turn, enables evidence-based decision-making and raises public trust. Lynch et al studied the public perception of newborn screening in Australia. Generally, the study participants welcomed the introduction of genomic sequencing into newborn screening programs. The main benefit was considered to be early diagnosis and the potential for early intervention and treatment, and informing expecting individuals and couples before childbirth was considered important (17). Most parents enrolled in the Babyseq project reported being very interested in receiving information on their baby's risk of developing a disease in childhood that can be prevented, treated, or cured (86.8%) and their risk of developing a disease during adulthood that can be prevented, treated, or cured (84.6%) (18). Peay et al developed and assessed an electronic system designed for public education on the topic of genomic sequencing in newborn screening. A great number of participants (>95%) stated that they had sufficient information and high trust regarding the program. The attitude toward the program was mostly positive, and almost all participants were satisfied they had participated (99%) (19).

The cost-effectiveness and economic impact of newborn screening expansion must also be assessed. Stark et al reported that changes in clinical management warranted by genomic sequencing results led to an average of 1578 Australian dollars saved per quality-adjusted life year gained, with no additional use of hospital services (20). On the other hand, when cascade testing and reproductive services were taken into account, the average was 8118 Australian dollars of additional cost per quality-adjusted life year gained (20). These two different results highlight the aspect of additional and ongoing downstream diagnostic/screening procedures that may be warranted to thoroughly investigate the significance of positive NBSeq results. This economic impact may potentially be mitigated by reaching a consensus regarding target gene lists with a view to the cost-effectiveness of early detection.

Widespread adoption at the population scale requires the establishment of parameters regarding the safe and effective use of NBSeq. The Paediatric Task Team of the Global Alliance for Genomics and Health's (GA4GH) Regulatory and Ethics Working Group reviewed current understanding and concerns regarding the use of genomic technologies for population-based newborn screening. They developed, by consensus, recommendations for clinicians, clinical laboratory scientists, and policy makers. The main point raised was the need to demonstrate the utility and cost-effectiveness of whole genome sequencing (WGS) in this regard, specifically addressing the issue of variant interpretation after testing. Second, a need for establishing locally-appropriate policy regarding disclosure of secondary and incidental findings, as well as ownership, appropriate storage, and access to genomic data was identified (21).

## Effectiveness of WGS technology for newborn sequencing

WGS is well-established as a scalable, high throughput, reliable technology. Sweeney et al used WGS to study a cohort of critically ill infants suffering from congenital heart disease. WGS alone was able to identify the respective variants five times more frequently than a combination of genetic panels and microarray. As such, WGS was more adept at providing actionable genomic findings (22).

From an NBSeq perspective, Kingsmore et al simulated newborn rapid WGS for a set of 388 treatable disorders in 454 707 UK Biobank subjects, and demonstrated a true positive rate of 89% and a negative predictive value of ≥99%. Their Delphi analysis revealed that for a proportion of these individuals, clinical signs and symptoms of their respective diseases could have been avoided to varying degrees if management had been instituted early in life. These results again show the utility of WGS in newborn screening programs (23).

Furthermore, dried blood spots (DBS) have been shown to be an acceptable sample type, thereby allowing to leverage well-established sample collection procedures already in use for traditional NBS, especially when considering deployment of WGS-based NBSeq at a public health scale. While the DNA yield of DBS was lower than that of EDTA blood samples, DBS-extracted DNA was of sufficient quality and quantity for PCR-free WGS, and all DBS samples yielded WGS that met quality control metrics for high-confidence variant calling (24). No difference was observed in the performance of DBS and peripheral-blood-extracted DNA in the detection of likely pathogenic gene variants in samples taken from individuals with cystic fibrosis or phenylketonuria (25).

A comprehensive meta-analysis by Nurchis et al comparatively assessed the cost-effectiveness of WGS and other genetic diagnostic methods. This was investigated on a database of 1600 publications, in the context of newborns and children with suspected genetic disorders. When comparing WGS to whole exome sequencing, an average net benefit of US$ 4073 was estimated in favor of WGS. When comparing WGS to clinical microarray, an average net benefit of 6003 dollars was estimated in favor of WGS (26). This analysis demonstrates the cost-effectiveness of WGS specifically in newborn sequencing, in comparison with more dated genetic diagnostic methods.

## Newborn sequencing considerations that need to be addressed

Genomic data interpretation poses complex challenges due to the vast amount of data generated, the presence of variants of uncertain significance, and the dynamic nature of our understanding of genetics. Advances in bioinformatics, machine learning, and genomic interpretation are crucial for the translation of genomic insights into actionable clinical insights. The fundamental criteria for gene selection are considered to be the age of onset, medical actionability, and validity of genotype-phenotype association. Despite these criteria being generally accepted as the principles for gene selection, the existing target gene lists for newborn sequencing are highly variable. Downie et al (27) curated a list of 55 consensus genes included by all six gNBS research projects they reviewed. The common identified reasons for the variability of NBSeq project gene lists included variable definitions of treatability and strength of gene-disease association (27). Similar efforts to curate ideal target genes for NBSeq are also ongoing. Another significant technological limitation arises from the fluid nature of our understanding of variants of uncertain significance, with increasing numbers being identified as more of an individual’s genomic data are analyzed.

Several other issues are also raised in a review by Parisi et al (28) as the actionability of genomic information across the lifespan is taken into account. First, the potential for genomic sequencing to discover adult-disease variants complicates the medical actionability of a genetic variant for a particular individual due to the potential need for long-term follow-up, especially for conditions with lower penetrance; even as it carries implications for cascade testing for family members, which may raise several ethical questions in and of itself. Second, a universal expansion of the newborn screening program would require more trained genetic counseling experts and other specifically educated health care providers. Third, diagnostic accessibility for such a universal program must be taken into account. Additionally, the expanded screening could be made optional and conducted in private institutions, which raises further ethical dilemmas (28).

An implementation challenge that screening programs generally face is the acceptability of the screening measure to the population being screened and their willingness to participate. Often a large target number of participants is required even in order for results to be considered scientifically valid and clinically relevant, which increases the barriers to implementation. Several researchers have studied participant engagement, recruitment, and retention through various forms of outreach. The Babyseq2 project has engaged with a Community Stakeholder Board to advise the project on several participant-facing aspects such as reports, return of results process, and survey design. In the EarlyCheck study based in North Carolina, USA, Paquin et al assessed the challenges in offering expanded newborn screening through a panel for rare disorders to new mothers. Recruitment letters achieved an enrollment rate of only 4%, while email notifications achieved a rate of 5% (29). Cope et al found that the return of normal results may be amenable to web-based portals, as the consequences to participants from not viewing results are modest at best, and the interpretation of a normal result is relatively straightforward (30).

The implications of racial and ethnic diversity must be taken into account when discussing the implementation of newborn sequencing. The Babyseq2 project is currently ongoing with the aim to enroll 500 infants from their primary care clinic, with at least half being from geographically and ethnically diverse backgrounds. Cakici et al evaluated discrepancies between ethnic groups in their enrollment in newborn genomic research studies and found that families speaking languages other than English or Spanish had an almost six times greater likelihood of refusing to participate in genomic diagnostic processes. This probability was somewhat reduced after the research staff received additional education. The authors concluded that enrollment was not as dependent on the ethnic group, as it was on the parents’ spoken language. This highlights the importance of improving the accessibility of these new technologies to patients and families with limited English-speaking abilities (31).

In order to ensure benefit from NBSeq, the adequacy of the health care provider workforce and therapeutic options for these patients must be carefully assessed. This is well supported in a review by Tesi et al, in which the authors state that clinical collaboration with geneticists, and education for all physicians about the concepts of genomics, are key to the integration of every aspect of personalized medicine (32). In light of the rapid availability of genomic sequencing in clinical medicine, Jenkins et al (33) evaluated the workforce of clinical geneticists across the US and concluded that there is a significant gap between the need and actual clinical geneticist workforce available. Policy decisions to educate more health care providers in genomic contexts continue to be necessary (33). An international advisory board convening in November 2021 highlighted the key role of medical geneticists in the successful deployment of gene therapies, as well as issued calls to action to improve workforce training and retention (34).

Additionally, one of the key determinants of the actionability of NBSeq results are the therapeutic options for the disease and their general accessibility. The increases in WGS accessibility and our knowledge regarding molecular mechanisms of genetic disorders have led to the development of many treatment options involving gene therapies (35). Gaviglio et al state that optimal development and distribution of gene-targeted therapies can be achieved through innovative approaches in research and clinical areas, as well as through the understanding of ethical, legal, and social implications (36). Another paper by Vockley et al recognizes the potential of WGS in newborn screening, as well as the bottleneck currently created by the slow pace of gene-targeted therapy development and treatment options in these patient groups (37).

Several studies have focused on exploring expert opinions on the population-scale implementation of NBSeq. A study published in 2021 by Bailey et al provided a set of twenty solutions regarding newborn sequencing modernization and integration, which experts then rated. A consensus was reached regarding the need for change in the newborn screening program considering the rapid development of novel therapies. The best-rated solutions were the establishment of universal data coordination mechanisms and networks of screening laboratories. Other well-rated solutions involved federal program alignment and the expansion of federal funding (38).

Andrews et al reported on a series of panel discussions involving newborn screening stakeholders, wherein the participants voted on the greatest barriers to the implementation of future newborn screening programs. The top three ranked issues were a lack of data informing decision-making, a greater burden on operating laboratories, and a significant period of time required for state-level implementation (39).

Thus, ethical aspects of consent, privacy, and the responsible use of genetic information must be carefully navigated to safeguard individual rights and maintain public trust. Implementation hurdles, including cost, infrastructure requirements, and the need for specialized expertise, also present barriers to the widespread adoption of NBSeq.

## Future directions

Data sharing will be a key resource in identifying and combating challenges, as well as amplifying the statistical power of all the research projects studying the medical, social, and economic factors affecting and resulting from NBSeq, thereby helping inform future policy. The International Consortium on Newborn Sequencing was formed in 2022 as “an alliance of genomic scientists and stakeholders who share a vision of responsibly implementing newborn sequencing,” with their stated mission being “to inform the clinical and public health research and implementation of genomic screening in newborns through the harmonization and aggregation of scientific evidence and resources.” (40,41).

## Conclusion

The feasibility of genomic sequencing in newborn screening has certainly been demonstrated in the literature. Many studies highlight the potential benefits of expanding screening programs by implementing this new approach. However, the existing hurdles cannot be denied and need to be addressed for such an innovative change to take place. Addressing these challenges requires a multidisciplinary approach involving collaboration among clinicians, researchers, policymakers, ethicists, and stakeholders across various sectors. Robust frameworks for informed consent, data protection, and governance are essential. Scalability and improving downstream health care access are vital for ensuring equitability, particularly in underserved communities and resource-limited settings. With dedication and concerted efforts, the integration of WGS into newborn screening holds the promise of revolutionizing early detection and intervention for treatable genetic disorders, ultimately leading to improved health outcomes for newborns worldwide.
